# Catalytic divergencies in the mechanism of L-arginine hydroxylating nonheme iron enzymes

**DOI:** 10.3389/fchem.2024.1365494

**Published:** 2024-02-08

**Authors:** Hafiz Saqib Ali, Sam P. de Visser

**Affiliations:** ^1^ Chemistry Research Laboratory, Department of Chemistry and the INEOS Oxford Institute for Antimicrobial Research, University of Oxford, Oxford, United Kingdom; ^2^ Manchester Institute of Biotechnology and Department of Chemical Engineering, The University of Manchester, Manchester, United Kingdom

**Keywords:** QM/MM, cluster models, enzyme catalysis, inorganic reaction mechanisms, iron enzymes, dioxygenases

## Abstract

Many enzymes in nature utilize a free arginine (L-Arg) amino acid to initiate the biosynthesis of natural products. Examples include nitric oxide synthases, which generate NO from L-Arg for blood pressure control, and various arginine hydroxylases involved in antibiotic biosynthesis. Among the groups of arginine hydroxylases, several enzymes utilize a nonheme iron(II) active site and let L-Arg react with dioxygen and *α*-ketoglutarate to perform either C_3_-hydroxylation, C_4_-hydroxylation, C_5_-hydroxylation, or C_4_−C_5_-desaturation. How these seemingly similar enzymes can react with high specificity and selectivity to form different products remains unknown. Over the past few years, our groups have investigated the mechanisms of L-Arg-activating nonheme iron dioxygenases, including the viomycin biosynthesis enzyme VioC, the naphthyridinomycin biosynthesis enzyme NapI, and the streptothricin biosynthesis enzyme OrfP, using computational approaches and applied molecular dynamics, quantum mechanics on cluster models, and quantum mechanics/molecular mechanics (QM/MM) approaches. These studies not only highlight the differences in substrate and oxidant binding and positioning but also emphasize on electronic and electrostatic differences in the substrate-binding pockets of the enzymes. In particular, due to charge differences in the active site structures, there are changes in the local electric field and electric dipole moment orientations that either strengthen or weaken specific substrate C−H bonds. The local field effects, therefore, influence and guide reaction selectivity and specificity and give the enzymes their unique reactivity patterns. Computational work using either QM/MM or density functional theory (DFT) on cluster models can provide valuable insights into catalytic reaction mechanisms and produce accurate and reliable data that can be used to engineer proteins and synthetic catalysts to perform novel reaction pathways.

## 1 Introduction

The 20 standard amino acids play crucial roles in biology and serve as fundamental building blocks in the design of many biological structures and natural products. They serve as both the constituents of macromolecular protein polymers and as platforms for the synthesis of small-molecule metabolites with functions related to biological defense, e.g., antibiotics and antifungal, or as signaling molecules in biosystems (Walsh, 2006; White and Flashman, 2016; Dunham and Arnold, 2020; Morita et al., 2021). In either role, specific modifications of these amino acids by enzymes contribute to the biochemical diversity essential for their various functions. For instance, a free L-tryptophan amino acid forms the starting point of the biosynthesis of the hormones serotonin and melatonin that function as neurotransmitters in the brain and trigger mood swings and happiness (Roberts and Fitzpatrick, 2013; Höglund et al., 2019). The conversion of a proline amino acid in a peptide chain to *R*-4-hydroxyproline by proline-4-hydroxylase enzymes enables crosslinking of collagen strands that gives them their strength (McDonough et al., 2006; Koski et al., 2009; Gorres and Raines, 2010). As a result, a major constituent of collagen is 4-hydroxyproline that plays a vital role in maintaining the integrity and resilience of various bodily tissues and organs in humans.

Many amino acids serve as essential building blocks in the biosynthesis of various antibiotics and natural products in microorganisms like bacteria and fungi (Bérdy, 2005; Liu et al., 2013; Herisse et al., 2020). A notable example of a natural amino acid used in a range of bioreactions in biology is L-arginine (L-Arg). This amino acid falls into the category of semi-essential or conditionally essential amino acids due to its synthesis by the body, which can vary depending on development stages, health conditions, or injuries (Barbul, 1986; Visek, 1986; Beaumier et al., 1996; Wu et al., 2009). The most common enzymatic use of L-Arg is within the group of nitric oxide synthase (NOS) enzymes, where L-Arg reacts on a heme center with dioxygen to form L-citrulline and NO (Stuehr, 1999; Groves and Wang, 2000). In the human body, NO has functions including blood pressure control through dilating blood vessels, as well as immunological functions. Other uses of L-Arg in biosystems include serving as a building block for the biosynthesis of a range of natural products with remarkable metabolic versatility, contributing to the synthesis of various compounds such as urea, ornithine, citrulline, creatine, agmatine, glutamate, proline, hydroxyls, and polyamines (Wu and Morris, 1998). Consequently, its metabolic processes are intricate and tightly regulated, which is unsurprising considering its diverse roles (Morris, 2002). The intricate nature of L-Arg metabolism stems not just from the array of enzymes engaged in its breakdown and its metabolites but also from their distinctive patterns of expression within cells (Morris, 2009).

**SCHEME 1:**
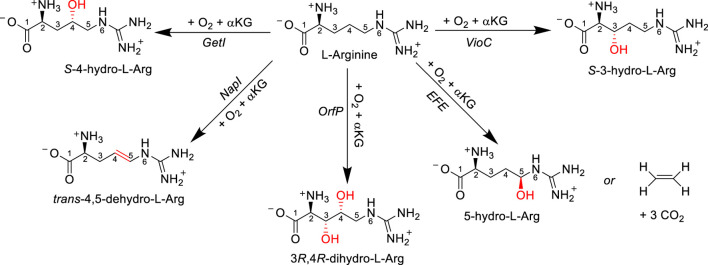
Enzymatic conversion of L-Arg by various Fe/*α*KG-dependent enzymes and obtained products.

One significant enzyme superfamily responsible for a substantial portion of known oxidative L-Arg modifications is the iron(II)- and *α*-ketoglutarate-dependent (Fe/*α*KG) oxygenase superfamily. In eukaryotic organisms, Fe/*α*KG enzymes primarily hydroxylate side chain functional groups of amino acids and mainly operate on protein or peptide substrates, serving structural or regulatory functions (Krebs et al., 2007; de Visser and Kumar, 2011; Martinez and Hausinger, 2015; Simaan et al., 2015; Herr and Hausinger, 2018). In contrast, in prokaryotes, Fe/*α*KG oxidants can target monomeric amino acids, leading to diverse reaction outcomes. A prominent example is clavaminate synthase (CAS), an *α*KG-dependent oxygenase (*α*KG is also called 2-oxoglutarate), which catalyzes sequential hydroxylation, oxidative cyclization, and desaturation reactions on an L-Arg derivative during the biosynthesis of the *β*-lactamase inhibitor known as clavulanic acid (Marsh et al., 1992). In several other antibiotic biosynthesis reactions, Fe/*α*KG oxidants play a crucial role in selectively hydroxylating or desaturating an L-Arg amino acid residue during the initial reaction step, highlighting the significance of enzyme activity in antibiotic production. For example, enzymes such as the viomycin biosynthesis enzyme VioC, nonribosomal peptide biosynthesis enzyme GetI, the streptothricin biosynthesis enzyme OrfP, and the naphthyridinomycin biosynthesis enzyme NapI all act on L-Arg. Despite the fact that all of these enzymes share >50% sequence similarity (Berman et al., 2000), they target different positions on the amino acid side chain and produce distinct products (Scheme 1). VioC selectively hydroxylates L-Arg at the C_3_ position within the viomycin pathway (Thomas et al., 2003; Barkei et al., 2009; Dunham et al., 2018a; Ali et al., 2021a), while GetI functions as a C_4_-hydroxylase of L-Arg (Zwick et al., 2019). OrfP is responsible for dihydroxylations at both the C_3_ and C_4_ positions of L-Arg in the streptothricin pathways (Chang et al., 2014; Ali et al., 2021b), and NapI performs the C_4_−C_5_ desaturation of L-Arg in the naphthyridinomycin pathway (Scott and Williams, 2002; Pu et al., 2013; Dunham et al., 2018b; Ali et al., 2023). Although the ethylene-forming enzymes (EFEs) allow the reaction of *α*KG on an iron(II) center with dioxygen to convert *α*KG into ethene and CO_2_ molecules, a side reaction leads to the hydroxylation of a free L-Arg at the C_5_ position (Martinez and Hausinger, 2016; Chaturvedi et al., 2021; Copeland et al., 2021; Yeh et al., 2022). It is unclear whether this L-Arg hydroxylation is part of the bioreaction or whether L-Arg binding is related to creating specific charge, electric dipole, and electric field perturbations to the active site (Chaturvedi et al., 2021). Nevertheless, the 5-hydroxyarginine product decomposes spontaneously into guanidine *L*-Δ^1^-pyrroline-5-carboxylate products.

**FIGURE 1:**
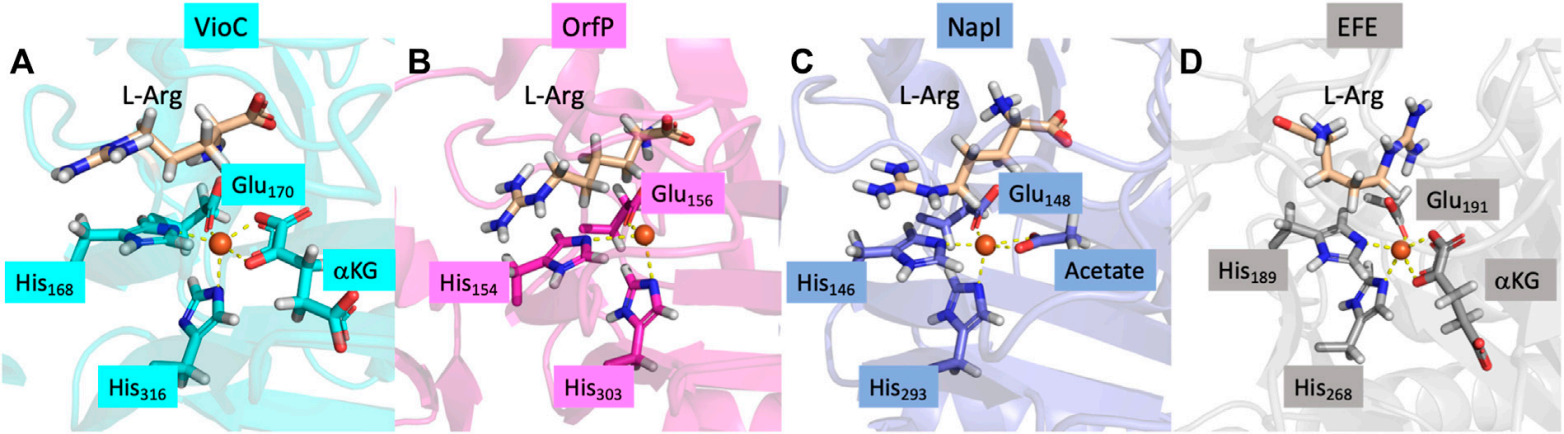
Crystal structure coordinates of **(A)** VioC (PDB ID: 6ALM), **(B)** OrfP (PDB ID: 4M2E), **(C)** NapI (PDB ID: 6DAW), and **(D)** EFE (PDB ID: 6VP4).

Structurally, Fe/*α*KG-dependent dioxygenases exhibit an iron(II) resting state, where the metal is bound to the protein via interactions involving the side chains of two histidine residues and either a carboxylate group from a Glu or Asp residue. The two residues located in the equatorial plane, one His residue and one carboxylate group of Glu or Asp, are typically separated by two residues within the protein loop, denoted as residues X and X + 2 along the protein chain (Berman et al., 2000; Ali et al., 2021a). An analysis of various protein structures of L-Arg-activating nonheme iron dioxygenases, retrieved from the Protein Data Bank (Berman et al., 2000), is shown in Figure 1 and contains both L-Arg and *α*KG. The structures reveal that the *α*KG group forms a bidentate ligand to iron(II), connecting through both the carboxylate and keto groups. Moreover, in these structures, it was observed that the terminal carboxylate group of *α*KG forms a salt bridge with the side chain of a preserved Arg residue. This Arg residue is on the same chain and positioned several residues away from the axial His residue in most *α*KG-dependent nonheme iron dioxygenases. The chain forms a loop around the *α*KG co-substrate that holds it in a specific orientation and position in the active site. Extracts of crystal structures of the selected L-Arg hydroxylases are shown in Figure 1 (Berman et al., 2000; Chang et al., 2014; Mitchell et al., 2017; Dunham et al., 2018b; Copeland et al., 2021).

**SCHEME 2:**
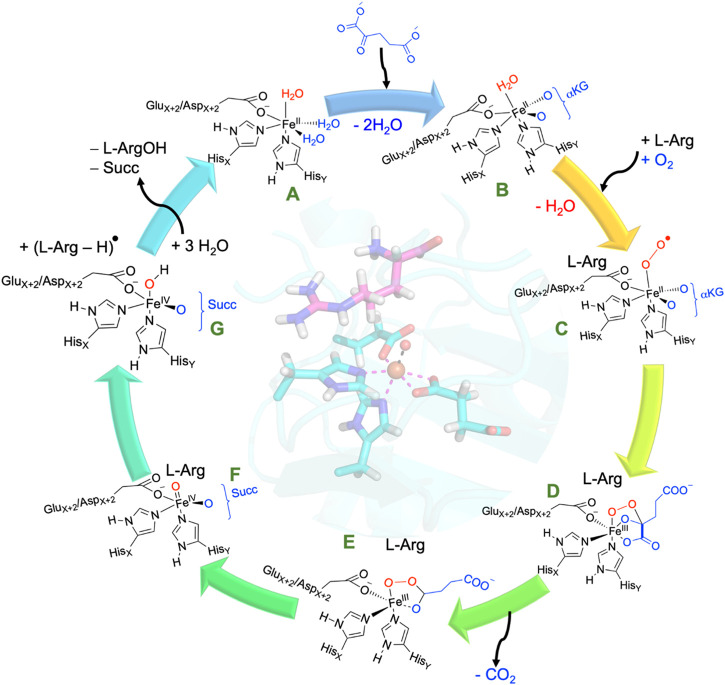
Consensus catalytic cycle of nonheme iron/*α*KG-dependent dioxygenases for L-Arg hydroxylation. *α*KG stands for *α*-ketoglutarate, Succ represents succinate.

All nonheme iron hydroxylases and halogenases follow similar initial catalytic steps, irrespective of their substrate or reaction type, whereby *α*KG in a reaction with dioxygen is converted into succinate and CO_2_ and generates a high-valent iron(IV)-oxo species, as shown in Scheme 2. The catalytic process initiates from the resting state (structure **A** in Scheme 2), where iron(II) is bound to the protein through the typical 2-His/1-carboxylate motif, and the remaining six ligand sites of the metal are occupied with water molecules. Most of the nonheme iron enzymes commonly feature a conserved, typical facial binding motif of 2-His/1-Glu or 2-His/1-Asp coordination of the iron(II), referred to as the facial triad (Berman et al., 2000; Bruijnincx et al., 2008; Kal and Que, 2017; de Visser et al., 2022). In VioC, these ligands are represented by His_168_, Glu_170_, and His_316_, while in NapI, these residues are His_146_, Glu_148_, and His_293_, as shown in Figure 1. Upon *α*KG binding (structure **B**), two water molecules are displaced from the metal and leaves only the water molecule trans to the axial histidine group, i.e., His_316_ in VioC. Subsequent binding of the L-Arg substrate results in the release of this remaining water molecule, creating space for O_2_ to bind and forming an end-on iron(III)-superoxo complex (structure **C**). The iron(III)-superoxo intermediate is transient and has never been directly observed, although there are implications in the ultraviolet–visible (UV-Vis) spectrum of cysteine dioxygenase for its existence (Tchesnokov et al., 2016). Moreover, computational models suggest that the terminal oxygen atom of the superoxo group attacks the *α*-keto position of *α*KG (Borowski et al., 2004; de Visser, 2007), thereby forming a bicyclic ring structure (structure **D**). Additionally, structure **D** is short-lived and possesses a weak C_1_−C_2_ bond in the *α*KG fragment that leads to its rapid disintegration into persuccinate through CO_2_ loss (structure **E**). However, the persuccinate bond is weak, and the O−O cleavage results in a coordinated oxygen atom, i.e., an iron(IV)-oxo (ferryl) intermediate (structure **F**) and a succinate dianion. The ferryl-oxo group is the active oxidant in the catalytic cycle that targets the substrate. In particular, in the hydroxylases and halogenases, the ferryl complex abstracts a hydrogen atom (H^•^) from the substrate, demonstrating significant reactivity even with very unreactive carbon centers. The resulting state contains a carbon-centered substrate radical (C^•^) and an iron(III)-hydroxo cofactor, which becomes a crucial turning point: its subsequent behavior determines the outcome of the reaction (structure **G**). Different pathways originating from this critical intermediate point result in the diverse and well-documented reaction outcomes within the Fe/*α*KG superfamily. The iron(IV)-oxo species has been trapped and characterized for various Fe/*α*KG-dependent dioxygenases through UV-Vis spectroscopy, electron paramagnetic resonance spectrometry, and Mössbauer spectroscopy (Krebs et al., 2007; Tchesnokov et al., 2016; Mitchell et al., 2017; Dunham et al., 2018a; Dunham et al., 2018b; Copeland et al., 2021). For taurine/*α*KG-dependent dioxygenase, even extended X-ray absorption fine structure (EXAFS) characterization that identified an Fe−O interaction of 1.62 Å has been reported (Riggs-Gelasco et al., 2004). In addition, resonance Raman studies with ^16^O_2_ and ^18^O_2_ established a difference spectrum and characterized the Fe−O vibration in the iron(IV)-oxo species at 821 cm^−1^ (Proshlyakov et al., 2004).

In hydroxylases after the hydrogen atom abstraction, the C^•^ radical attacks the hydroxyl group of the iron(III)-hydroxo complex and forms a new C−O bond (Huang and Groves, 2017; Gérard et al., 2022). This OH rebound step creates the alcohol product complex and regenerates the iron(II) cofactor for subsequent cycles. Products (hydroxylated arginine and succinate) are released, and the metal ligand positions filled with water molecules to return to the resting state of the catalytic cycle. The radical coupling step, known as oxygen rebound, is generally not rate-determining as kinetic isotope effect measurements obtained a large rate constant change when hydrogen atoms in the substrate are replaced by deuterium atoms (Krebs et al., 2007; Dunham et al., 2018a; Dunham et al., 2018b). Indeed, the radical intermediate C^•^/Fe(III)-OH state has never been characterized experimentally and is transient in Fe/*α*KG hydroxylases, and hence, its lifetime must be short. However, for some reaction mechanisms (*vide supra*), the radical intermediate leads to bifurcation processes where two reaction channels are possible. Often an intricate balance between the structure, orientation, and local charge distributions determines the ultimate product distributions. In some cases, the oxygen rebound process can be significantly limited, possibly due to certain enzymes having a distinct geometric arrangement in the ferryl complex, allowing for this suppression. For example, the desaturation of L-Arg by NapI enzymes, involving Fe/*α*KG, is influenced by the orientation of substrate binding and the polarity, along with hydrogen bonding interactions within the substrate-binding pocket (Ali et al., 2023). These factors guide the reaction toward desaturation products rather than hydroxylation by stabilizing the position of the ferryl complex.

All Fe(II)/*α*KG-dependent dioxygenases are expected to undergo a catalytic cycle, as shown in Scheme 2, where *α*KG with dioxygen is converted into succinate, CO_2_, and an iron (IV)-oxo species. However, as many of the proposed intermediates in the cycle remain elusive, most evidence on the catalytic cycle comes from computational modeling. Calculations at various levels of theory (Borowski et al., 2004; de Visser, 2007; Wójcik et al., 2016; Ghafoor et al., 2019; de Visser et al., 2022) reported on the reaction steps leading to the iron(IV)-oxo species and generally give low free energies of activation of approximately 10 kcal mol^−1^ for the step between structures **C** and **D** and lower barriers for all other reaction steps leading to the iron(IV)-oxo species. As such, it is expected that dioxygen binding will rapidly lead to a highly stable iron(IV)-oxo species. The next step of the reaction, therefore, and particularly the second-coordination sphere interaction of the substrate and oxidant, determines the selectivity patterns (de Visser, 2020; Wojdyla and Borowski, 2022). In this review paper, we will summarize and compare substrate activation processes by the iron(IV)-oxo species of Fe(II)/*α*KG-dependent dioxygenases and compare several L-Arg-activating enzymes. We ask ourselves how these enzymes direct their chemoselectivity to the required position of the substrate and block alternative reaction products. Our computational studies focused on uncovering how these enzymes activate different C−H bond positions of the same L-Arg substrate and thereby yield a diverse array of hydroxylated and desaturation products. Through a comprehensive analysis of the structures, electronic properties of the substrate, oxidant, and second-coordination sphere, we established the key factors that drive the reactions into a specific direction and unravel the nuances underlying these differences in product formation.

## 2 Mechanism of L-arginine activation by Fe(II)/*α*KG-dependent dioxygenases

Many researchers have investigated the catalytic cycle of iron(II)/*α*KG-dependent dioxygenases, and there are reports on spectroscopic characterization including UV-Vis absorption, electron paramagnetic resonance spectrometry, resonance Raman spectroscopy, Mössbauer spectroscopy, and EXAFS measurements on various catalytic cycle intermediates in a range of isozymes (Proshlyakov et al., 2004; Riggs-Gelasco et al., 2004; Krebs et al., 2007; Martinez and Hausinger, 2015). However, since many proposed intermediates are short-lived, experimental work on the catalytic cycle of these enzymes is challenging. Often, insights into reaction pathways and the nature of the short-lived species can be gained only through computational studies. The computational approaches generally range from molecular dynamics studies on full enzymatic systems to quantum mechanics/molecular mechanics (QM/MM) on an enzyme structure. Thus, in QM/MM (Senn and Thiel, 2007a; Senn and Thiel, 2007b; Quesne et al., 2016; Hofer and de Visser, 2018), a complete enzyme with co-factors, substrate, and a water layer is selected, whereby the inner core of the system, i.e., the active site, is calculated with a QM method, and the rest of the protein and solvent, with a MM forcefield. This approach keeps the long-range interactions between the QM and MM regions and restricts motions of the active site atoms during geometry optimization. An alternative approach is using density functional theory (DFT) cluster models of size of up to approximately 500 atoms (Siegbahn and Blomberg, 2010; Sheng et al., 2020; Himo and de Visser, 2022). In DFT cluster models, the active site and second-coordination sphere are calculated with an accurate DFT approach although sometimes some protein atoms are fixed to keep the structure close to the protein model the calculations started from. In Ali and de Visser (2022), we showed that both approaches can reproduce experimentally determined free energies of activation of enzymatic systems to within 2 kcal mol^−1^; however, either a large QM region in QM/MM or a cluster model with more than 250 atoms is needed to achieve this accuracy. In particular, the second-coordination sphere interactions including hydrogen bonding and dipole moment interactions appear crucial for the correct description of the catalysis reaction. A similar level of accuracy was obtained for the calculations of barrier heights of the oxygen atom transfer of biomimetic models, as compared to the experimental work (Cantú Reinhard et al., 2017; Mukherjee et al., 2019). Using cluster models, our groups also explored regioselectivities and pathways, leading to by-products (Timmins et al., 2017; Lin et al., 2022; Mokkawes and de Visser, 2023), and showed that these bifurcation pathways differ by only a few kcal mol^−1^ in some cases; hence, modeling will need to predict the correct ordering and able to deal with these small energy differences. The DFT calculations on large cluster models reproduce experimental product distributions very well, and hence, the systematic errors of the DFT approaches do not appear to influence the predictions of reaction mechanisms. Consequently, computational modeling using cluster models is suitable for calculations on complicated reaction channels with small energy differences.

The consensus catalytic mechanism, as described above in Scheme 2, entails dioxygen binding to the iron center, followed by decarboxylation of *α*KG, resulting in succinate and the creation of an iron(IV)-oxo compound. This iron(IV)-oxo species is considered the primary oxidizing agent but short-lived. Recent computational studies from our group on L-Arg activation by Fe(II)/*α*KG dioxygenases show that the L-Arg transformation to hydroxylated or desaturated products is influenced by how the substrate binds, the intrinsic electric field effect in the protein pocket, and the hydrogen bonding interactions between the substrate and its direct environment within the substrate-binding pocket (Ali et al., 2021a; Ali et al., 2021b; Ali et al., 2023).

**FIGURE 2:**
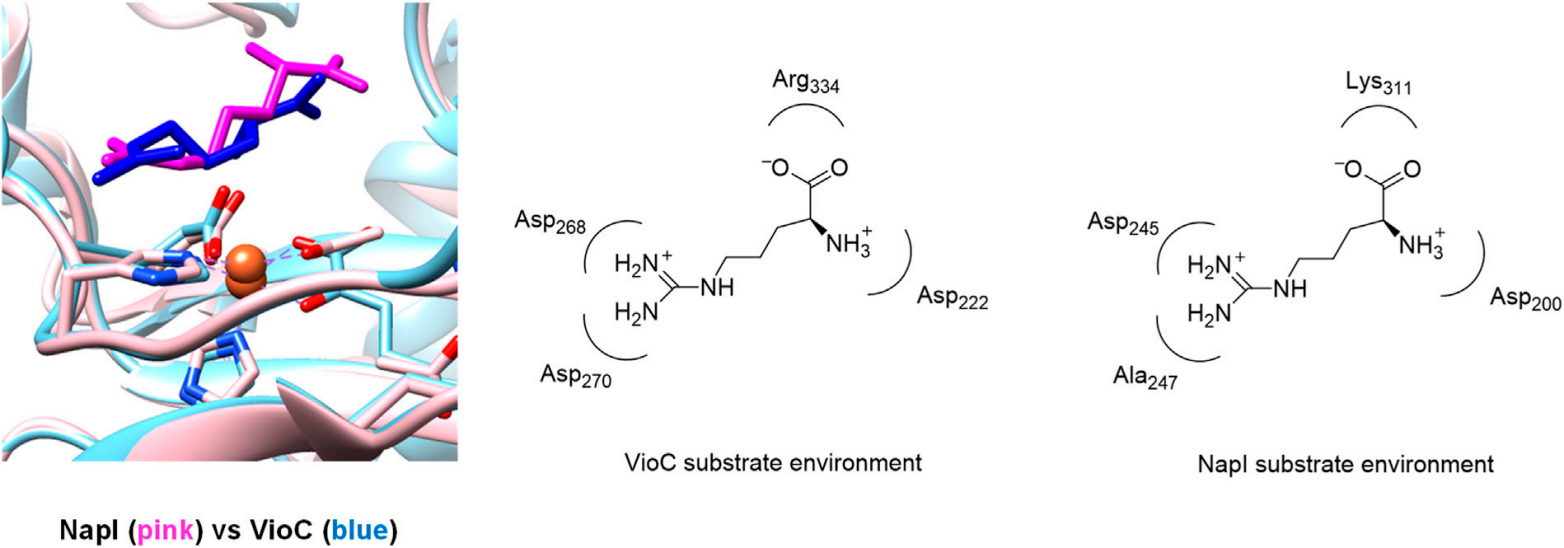
Overlay of the crystal structure coordinates of VioC (PDB ID: 6ALM) and NapI (PDB ID: 6DAW).

**SCHEME 3:**
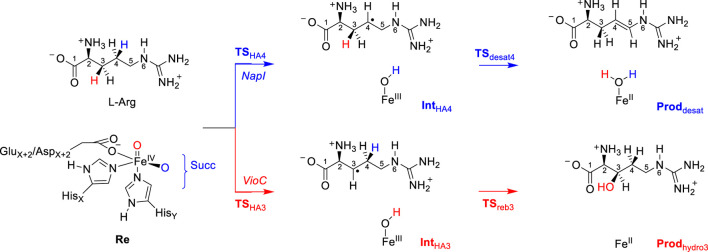
Radical reaction mechanism of L-Arg hydroxylation by VioC and L-Arg desaturation by NapI.

Figure 2 shows an overlay of the crystal structure coordinates of the L-Arg-bound iron(II) complexes of NapI and VioC, as taken from the 6ALM and 6DAW pdb files (Berman et al., 2000; Mitchell et al., 2017; Dunham et al., 2018b). As can be seen, both enzymes display the characteristic 2-His/1-carboxylate iron(II) coordination with the groups in similar positions. In addition, both enzymes bind *α*KG through the carboxylate and *α*-keto groups to the iron. Substrate L-Arg is shown in Figure 2 as well, and as can be seen, it is located in a similar orientation and position in NapI and VioC, yet VioC gives substrate C_3_-hydroxylation, while NapI reacts through desaturation of the C_4_−C_5_ bond. As such, the crystal structure coordinates do not give a clear view on the origin of the regioselectivity of these enzymes. The right-hand side of Figure 2 shows nearby residues from the substrate in NapI and VioC. Not surprisingly, the carboxylate group of the substrate interacts with a positively charged amino acid into a salt bridge, namely, with Arg_334_ in VioC and with Lys_311_ in NapI. The positively charged ammonium group of the substrate interacts with a carboxylate side chain of an Asp residue in both enzymes. The major difference in substrate binding between the two enzymes resides in the interactions of the guanidinium group of L-Arg with the protein. Thus, in VioC, it interacts with two carboxylate groups, namely, the side chains of Asp_268_ and Asp_270_. By contrast, NapI only has one carboxylate group in that region, namely, Asp_245_, and the residue with number 247 is not an Asp/Glu amino acid but Ala. Consequently, there are differences in binding interactions of the substrate in the two isozymes, whereby in VioC, the substrate will be more strongly bound with an additional salt bridge from one extra Asp residue. Moreover, since Asp is negatively charged, these differences in the substrate-binding pocket between VioC and NapI will incur changes in the local dipole moment, charge distributions, and local electric field patterns that may influence reactivities. To understand whether these seemingly minor differences in substrate positioning and second-coordination sphere influence catalysis and product distributions, a series of computational studies were performed on the various enzyme models. The mechanisms focused on the reaction pathways starting from an iron(IV)-oxo species with nearby L-Arg bound, i.e., a reactant complex **Re**, as explained in Scheme 3. Thus, the iron(IV)-oxo species abstracts a hydrogen atom from the substrate to form an iron(III)-hydroxo species. In VioC, this hydrogen atom abstraction is from the C_3_−H bond (bottom pathway in Scheme 3), whereas in NapI, the abstraction is from the C_4_−H group (top channel in Scheme 3). Normally, hydrogen atom abstraction is followed by OH rebound to form the alcohol product complexes (Latifi et al., 2009; Sahu et al., 2014; Huang and Groves, 2017; Timmins et al., 2018a; Gérard et al., 2022); however, in some cases, a second hydrogen atom abstraction is possible to give desaturation reactions (Kumar et al., 2009), as is the case in NapI.

**FIGURE 3:**
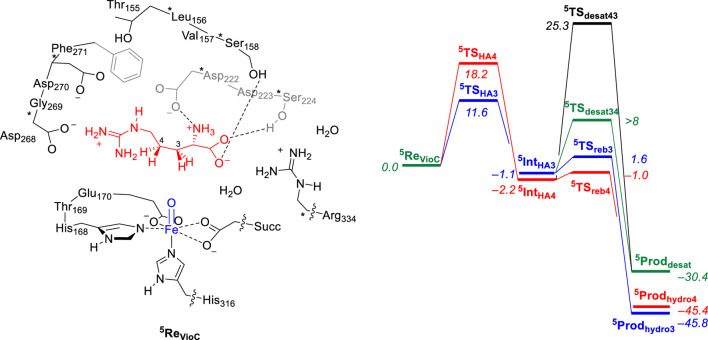
Relative free energy (ΔG) landscape for L-Arg hydroxylation at the C_3_ and C_4_ positions and desaturation across the C_3_−C_4_ bond by an iron(IV)-oxo species of a VioC model complex using calculations on the cluster model shown on the left. Free energies are expressed in kcal mol^−1^ and represent energies as obtained with UB3LYP-D3/BS2//UB3LYP/BS1 with solvent, zero-point energies, and thermal and entropic corrections included at 298 K. BS1 refers to LANL2DZ with core potential on iron and 6-31G* on the rest of the atoms, while a BS2 basis set has LACV3P+ with core potential on iron and 6-311+G* on the rest of the atoms. Data can be obtained from Ali et al. (2021a).

Let us first start with a description of the calculated reaction mechanism of L-Arg activation by VioC and the possible mechanisms leading to C_3_-hydroxylation, C_4_-hydroxylation, and C_3_−C_4_ desaturation. In particular, the viomycin biosynthesis enzyme VioC selectively hydroxylates L-Arg, specifically at the C_3_ position during the biosynthesis of its antibiotic, and there is no evidence of C_4_-hydroxylation or C_3_−C_4_ desaturation byproducts (Thomas et al., 2003; Yin and Zabriskie, 2004; Barkei et al., 2009; Helmetag et al., 2009; Mitchell et al., 2017; Dunham et al., 2018a). Figure 3 provides a detailed depiction of the energy landscape for the reaction pathways leading to the various intermediates and products in the process. The activation of L-Arg initiates from the iron(IV)-oxo species and commences by abstracting a hydrogen atom either from the C_3_ position or an adjacent site, such as the C_4_ position of the substrate via the transition states ^5^ **TS**_HA3_ and ^5^ **TS**_HA4_, respectively. Both of these steps lead to an iron(III)-hydroxo species with a substrate radical on either substrate atom C_3_ (^5^ **Int**_HA3_) or on C_4_ (^5^ **Int**_HA4_). The abstraction of a hydrogen atom from the C_3_−H bond necessitates a free energy of activation of ΔG^‡^ = 11.6 kcal mol^−1^ within the quintet spin state. By contrast, the abstraction of a hydrogen atom from the C_4_−H position of L-Arg by the VioC model presents a notably higher barrier than C_3_−H abstraction, registering approximately ΔG^‡^ = 18.2 kcal mol^−1^ within the quintet spin state. Interestingly, the formation of the radical intermediates ^5^ **Int**_HA3_ and ^5^ **Int**_HA4_ releases almost the same amount in energy, and ^5^ **Int**_HA3_ was located at ΔG = −1.1 kcal mol^−1^, while the C_4_−H radical intermediate ^5^ **Int**_HA4_ is slightly more stable at ΔG = −2.2 kcal mol^−1^. Therefore, the calculations predict a different ordering for the thermodynamics of the reaction (^5^ **Int**_HA3_ vs. ^5^ **Int**_HA4_), as compared to the kinetics (^5^ **TS**_HA3_ vs. ^5^ **TS**_HA4_). This phenomenon is called negative catalysis and means that the protein disrupts the kinetics through the second-coordination sphere effect so that the Bell–Evans–Polanyi principle does not apply, and the product distribution will not be based on the most thermodynamically favorable pathway (Ghafoor et al., 2019; de Visser et al., 2021). However, despite this, calculations indicate that products primarily originate from C_3_−H atom abstraction. This aligns with experimental findings of VioC, showcasing predominant 3-hydroxyarginine products, consistent with the observed dominance of C_3_-hydroxylation products for L-Arg as a substrate.

In the catalytic cycle’s subsequent phase, the hydroxyl group from the iron(III)-hydroxo species either rebounds to the radical and forms the alcohol products or gathers another hydrogen atom to create water and 3,4-dehydroarginine products (^5^ **Prod**_desat_). Transition states were characterized for OH rebound to the C_3_ and C_4_ positions from ^5^ **Int**_HA3_ and ^5^ **Int**_HA4_, respectively, namely, the structures ^5^ **TS**_reb3_ and ^5^ **TS**_reb4_ that give 3-hydroxyarginine (^5^ **Prod**_hydro3_) and 4-hydroxyarginine (^5^ **Prod**_hydro4_) products. In addition, we located a transition state for hydrogen atom abstraction from the C_3_−H position from ^5^ **Int**_HA4_, namely, ^5^ **TS**_desat43_, but were unsuccessful in fully characterizing the transition state for hydrogen atom abstraction from the C_4_−H position from ^5^ **Int**_HA3_, i.e., ^5^ **TS**_desat34_. Nevertheless, a constraint geometry scan for this pathway shows that it is well higher in energy than ^5^ **TS**_reb3_, and consequently, OH rebound should be the dominant pathway. Therefore, for the VioC model, the calculations show that desaturation is unfavorable over the OH rebound, regardless of whether the initial hydrogen atom abstraction proceeds from the C_3_−H or C_4_−H bonds. In addition, the work indicates a significantly higher free energy of activation for hydrogen atom abstraction from the C_4_−H bond than that from the C_3_−H group. Overall, the OH rebound to form 3-hydroxyarginine products is dominant, and the calculations predict little or no desaturation products. Similarly, intermediate ^5^ **Int**_HA4_ shows a clear preference for the rebound pathway over desaturation, aligning with experimental product distributions that favor C_3_-hydroxylation by VioC. The computational studies highlight that the desaturation pathway faces a notably high second hydrogen atom abstraction barrier that makes it unlikely for VioC to induce L-Arg desaturation. This emphasizes the dominance of the OH rebound mechanism in the enzymatic reaction process.

**FIGURE 4:**
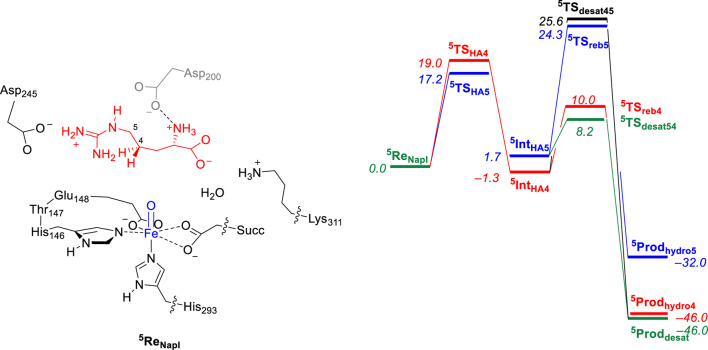
QM/MM calculated relative free energy (ΔG) landscape for L-Arg hydroxylation at the C_4_ and C_5_ positions and desaturation across the C_4_−C_5_ bond by an iron(IV)-oxo species of a NapI model complex. The QM region is shown on the left. Free energies are expressed in kcal mol^−1^ and represent energies as obtained with UB3LYP-D3/BS2//UB3LYP/BS1 with solvent, zero-point energies, and thermal and entropic corrections included at 298 K. BS1 refers to LANL2DZ with core potential on iron and 6-31G* on the rest of the atoms, while a BS2 basis set has LACV3P+ with core potential on iron and 6-311+G* on the rest of the atoms. Data can be obtained from Ali et al. (2023).

In a subsequent computational study (Ali et al., 2023), we used the QM/MM approach and set up a full enzyme model of NapI and calculated the L-Arg reaction with the iron(IV)-oxo species to form 4,5-dehydroarginine, 4-hydroxyarginine, and 5-hydroxyarginine products, as shown in Figure 4. A QM region was selected that contains the metal and its first-coordination sphere, the substrate, and its polar second-coordination sphere residues, namely, the side chains of Asp_200_, Asp_245_, and Lys_311_. Subsequently, local minima and transition states were characterized for the mechanism, leading to the various products. Similarly, in line with the work on VioC, the hydrogen atom abstraction step is rate-determining and ΔG^‡^ = 17.2 kcal mol^−1^ for abstraction of the C_5_−H atom is calculated, while the C_4_−H bond requires an activation energy of ΔG^‡^ = 19.0 kcal mol^−1^. The latter is close to the value obtained for VioC, and therefore, it appears that the second-coordination sphere does not affect the C_4_−H barrier dramatically. Furthermore, for NapI, the reaction proceeds through negative catalysis, whereby the ordering of the radical intermediates is different from that of the transition states. As such, NapI will react dominantly through C_5_−H hydrogen atom abstraction to form the iron(III)-hydroxo with C_5_ radical (^5^ **Int**_HA5_). From both radical intermediates, we calculated the desaturation pathway, where a second hydrogen atom abstraction gives a double bond along C_4_−C_5_, as well as a pathway for the OH rebound. From ^5^ **Int**_HA5_, the lowest barrier is hydrogen atom abstraction from the C_4_−H group with a barrier of ΔG = 6.5 kcal mol^−1^ above ^5^ **Int**_HA5_. By contrast, the OH rebound barrier is high in energy and found to be more than 16 kcal mol^−1^ higher than the hydrogen atom abstraction barrier. Consequently, NapI will react to form 4,5-dehydroarginine products through sequential hydrogen atom abstraction from the C_5_−H and C_4_−H bonds. Interestingly, if the reaction starts with hydrogen atom abstraction from the C_4_−H bond, then the iron(III)-hydroxo and C_4_-radical does not form the desaturated products but will result in C_4_-hydroxylation instead due to a lower rebound barrier. To understand the differences between the two isozymes and why they give different product distributions, we analyzed all data in detail and particularly focused on the electron transfer steps and electronic configurations of the various intermediates and the local environment around the substrate and oxidant.

**FIGURE 5:**
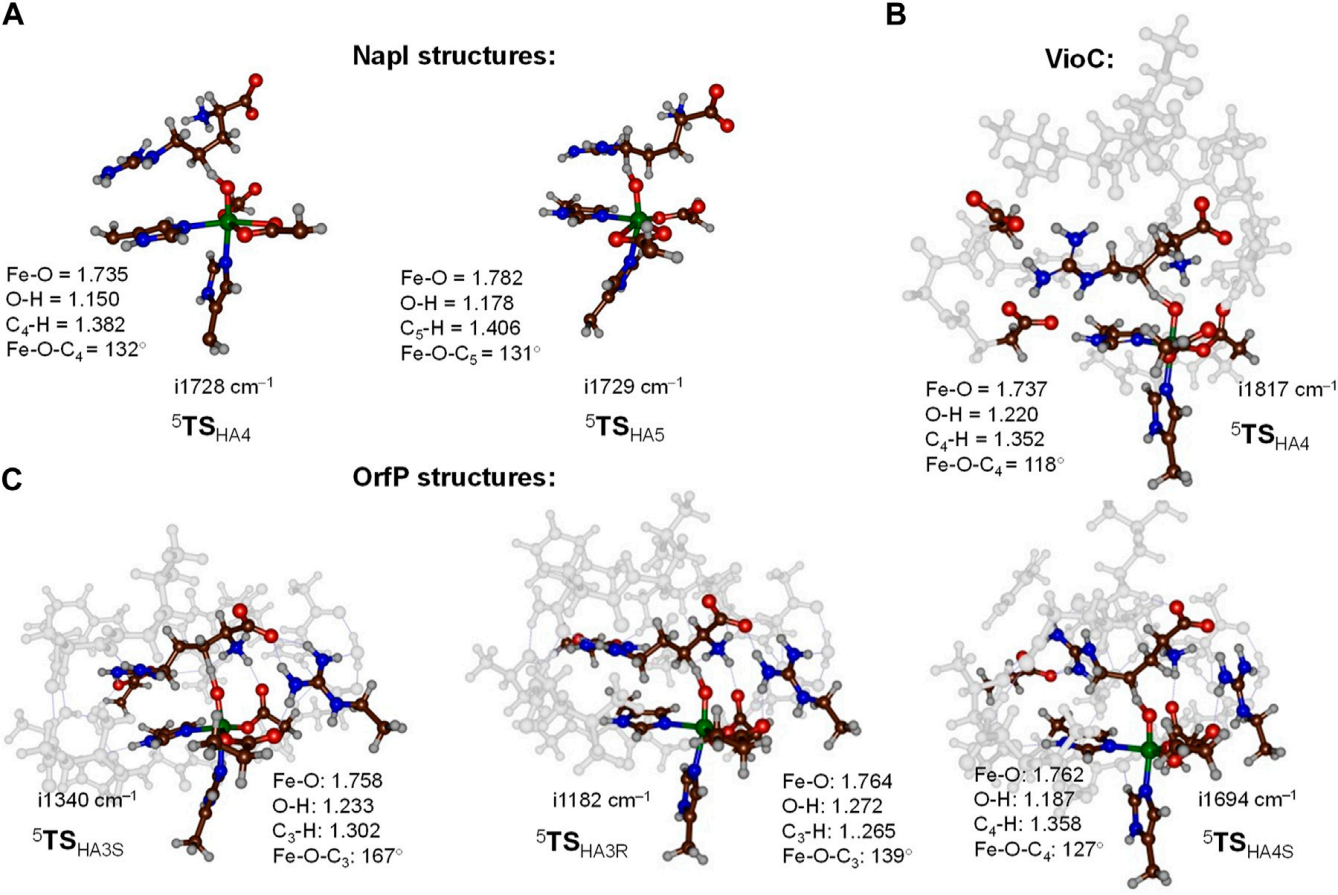
Optimized transition state structures for hydrogen atom abstraction steps from L-Arg by NapI (part **(A)**), VioC (part **(B)**), and OrfP (part **(C)**) enzymes with bond lengths in Å and angles in degrees. **(A)** QM/MM (UB3LYP/BS1:Amber) optimized transition states for NapI. **(B, C)** DFT cluster model calculated at the UB3LYP/BS1 level of theory.

Optimized transition state geometries for hydrogen atom abstraction from the C_4_−H and C_5_−H bonds of L-arginine by NapI, VioC, and OrfP models are shown in Figure 5 with data taken from Ali et al. (2021a); Ali et al. (2021b); Ali et al. (2023). All transition states have a large imaginary frequency of well over i1000 cm^−1^, which implies that the reaction will proceed with a large amount of quantum chemical tunneling and will incur a large kinetic isotope effect when the transferring hydrogen atoms are replaced by deuterium atoms (Barman et al., 2019). The three C_4_−H hydrogen atom abstraction transition states have similar bonding interactions and the Fe−O distances that range from 1.735 to 1.762 Å. This is the result of the same electronic configuration for all structures due to the same electron transfer patterns; see next section for details. In addition, the C_4_−H and O−H distances in the **TS**_HA4_ transition states are alike, whereby all three transition state structures have a product-like geometry with shorter O−H than C_4_−H distances. The only major difference between the three transition state structures for C_4_−H abstraction refers to the position of the substrate, which causes a change in the Fe−O−C_4_ angle from 118° in the VioC model to 132° in the NapI system. Despite the differences in the angle, the VioC and NapI transition states for hydrogen atom abstraction from the C_4_−H position are of similar energy. Interestingly, the optimized transition state structure for C_5_−H hydrogen atom abstraction (^5^ **TS**_HA5_) for NapI is not significantly different from that for C_4_−H hydrogen atom abstraction with similar distances and angles. As such, the preference of C_5_−H hydrogen atom abstraction does not appear from the optimized geometries, which encouraged us to analyze the environment and the fundamental factors for hydrogen atom abstraction.

The work on L-Arg activation by OrfP investigated the hydrogen atom abstraction of both hydrogen atoms on the C_3_−H position, leading to the formation of stereoisomers, namely, the *R*-3-hydroxyarginine and *S*-3-hydroxyarginine products. Both *pro*-*R* and *pro*-*S* transition state structures (^5^ **TS**_HA3R_ and ^5^ **TS**_HA3S_) are displayed alongside each other in Figure 5. Structurally, they are very similar, and the only major difference relates to the Fe−O−C_3_ angle that is 167° in the *pro*-*S* transition state and 139° in the *pro*-*R* transition state. These differences are related to the position of these hydrogen atoms in the structure, but energetically, the barriers are ΔE + ZPE = 12.1 kcal mol^−1^ for ^5^ **TS**_HA3S_ and ΔE + ZPE = 15.5 kcal mol^−1^ for ^5^ **TS**_HA3R_. The calculations show that substrate positioning will drive a stereo- and regioselective reaction mechanism, whereby certain C−H bonds become preferred for cleavage by the iron(IV)-oxo species.

## 3 Electron transfer during L-arginine activation by Fe(II)/*α*KG-dependent dioxygenases

**FIGURE 6:**
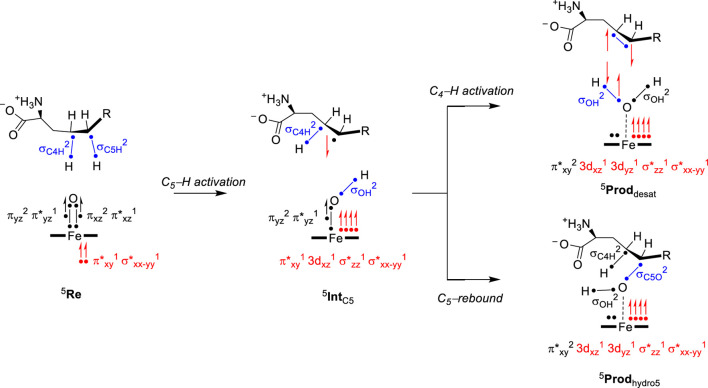
Electron transfer pathways and change in electronic configuration and the oxidation state of intermediates during L-Arg desaturation and hydroxylation in NapI.

To highlight the electronic configuration changes during the substrate desaturation and hydroxylation mechanisms for L-Arg-activating nonheme iron(IV)-oxo species, we show a valence bond diagram that focuses on the valence states of the substrate and oxidant. We used these valence bond diagrams previously to understand bifurcation pathways and selectivity mechanisms (Faponle et al., 2016; Timmins et al., 2018b; Ali et al., 2020). Thus, as experimentally determined (Krebs et al., 2007), the iron(IV)-oxo species of nonheme iron hydroxylases is in a quintet spin state. Electronically, the metal 3d orbitals mix with first-coordination sphere ligands and split into bonding and antibonding orbitals. The valence orbitals are labeled as π*_xy_, π*_xz_, π*_yz_, σ*_x2-y2_, and σ*_z2_ molecular orbitals and contain a dominant 3d contribution on the metal. The σ*_z2_ orbital is virtual and represents the antibonding interaction of the 3d_z2_ iron orbital with 2p_z_ orbitals on the axial nitrogen atom of the His residue and the oxo group. The π*_xy_ and σ*_x2-y2_ orbitals are in the xy–plane and represent antibonding interactions of the metal with the equatorial ligands, namely, the His, Asp, and succinate groups. Finally, the π*_xz_ and π*_yz_ orbitals are part of two 2-center-3-electron bonds along the Fe−O group for the antibonding interactions between the 3d iron orbitals with 2p orbitals on the oxo group. Overall, the iron(IV)-oxo species has electronic configuration of *π*_xz_^2^ π*_xz_^1^ *π*_yz_^2^ π*_yz_^1^ π*_xy_^1^ σ*_x2-y2_^1^ in a quintet spin state. Interestingly, biomimetic models with 6-coordination often give a triplet spin ground state, while the enzymatic structure has 5-coordination and a high-spin state (Latifi et al., 2013). In the valence bond diagram (Figure 6), we identify a chemical bond as two dots with a line in between them, while unpaired electrons are given as a separate dot with the spin direction (*α* or *β*-spin) shown with a single-headed arrow.

During the hydrogen atom abstraction, the C−H bond of the substrate is broken, i.e., the C_5_−H orbital (*σ*_C5H_) in Figure 6. The hydrogen then forms a new O–H orbital (*σ*_OH_) with the oxo group of the oxidant. This step generally leads to elongation of the Fe−O bond and the cleavage of the *π*_xz_ and π*_xz_ molecular orbitals. One of those electrons moves into the *σ*_OH_ bond, while one stays behind in the nonbonding 3d_xz_ orbital with dominant metal contribution. The last electron from the 3-electron *π*_xz_/π*_xz_ set of orbitals is promoted into the virtual σ*_z2_ orbital. The thus obtained electronic configuration of the radical intermediate with *σ*_OH_^2^ 3d_xz_^1^ *π*_yz_^2^ π*_yz_^1^ π*_xy_^1^ σ*_x2-y2_^1^ σ*_z2_^1^ configuration is called the *σ*-pathway for hydrogen abstraction. The alternative mechanism is called the *π*-pathway, where instead of an up-spin electron transfer from substrate into σ*_z2_, a down-spin electron is moved into π*_xy_. The latter gives an electronic configuration of *σ*_OH_^2^ 3d_xz_^1^ *π*_yz_^2^ π*_yz_^1^ π*_xy_^2^ σ*_x2-y2_^1^ σ*_z2_^0^ and has the radical on the substrate as down-spin, while it is up-spin in the *σ*-pathway (de Visser, 2006; Hirao et al., 2011; Tang et al., 2012; Bernasconi and Baerends, 2013; Usharani et al., 2013; Ye et al., 2013; Quesne et al., 2014; Mukherjee et al., 2021). Typically, the *σ*-pathway is energetically and kinetically favored over the *π*-pathway, but often, they are close in energy. Structurally, the electron transfer into σ*_z2_ often gives a substrate attack from the top with a large Fe−O−C angle (close to 180°), while in the *π*-pathway, the substrate attacks more under and angle with typical Fe−O−C angles of approximately 120° (Hirao et al., 2011). The reaction kinetics hinge on the electron transfer guided by substrate and oxidant positioning, which needs to be accommodated for in the substrate-binding pocket of the enzyme. All transition states for hydrogen atom abstraction in L-Arg-activating nonheme iron dioxygenases, namely, VioC, NapI, and OrfP, were found to proceed through the same electron transfer processes, where a *σ*-pathway was followed, leading to a radical intermediate. Consequently, the differences in reactivity have no electronic basis but appear to be the result of substrate and oxidant positioning and their second-coordination sphere.

After the radical intermediate, either another hydrogen atom abstraction takes place or OH rebound occurs to form the alcohol product complex. Both product complexes have the same electronic configuration of an iron(II) atom coupled to a closed-shell product. In particular, the product electronic configuration is π*_xy_^2^ 3d_xz_^1^ 3d_yz_^1^ σ*_z2_^1^ σ*_x2-y2_^1^ and results from the cleavage of the *π*_yz_/π*_yz_ 3-electron bond into atomic orbitals. The 3d_yz_ orbital is now a nonbonding orbital with one electron, while another electron from the *π*_yz_/π*_yz_ orbitals is transferred to σ*_z2_ (*π*-pathway) or π*_xy_ (*σ*-pathway). The final electron originating from the *π*_yz_/π*_yz_ set of orbitals forms the *σ*_OH_ orbital (in the desaturation pathway) or the *σ*_C5O_ orbital (in the OH rebound).

## 4 Environmental effects on bifurcation pathways for L-arginine activation

**FIGURE 7:**
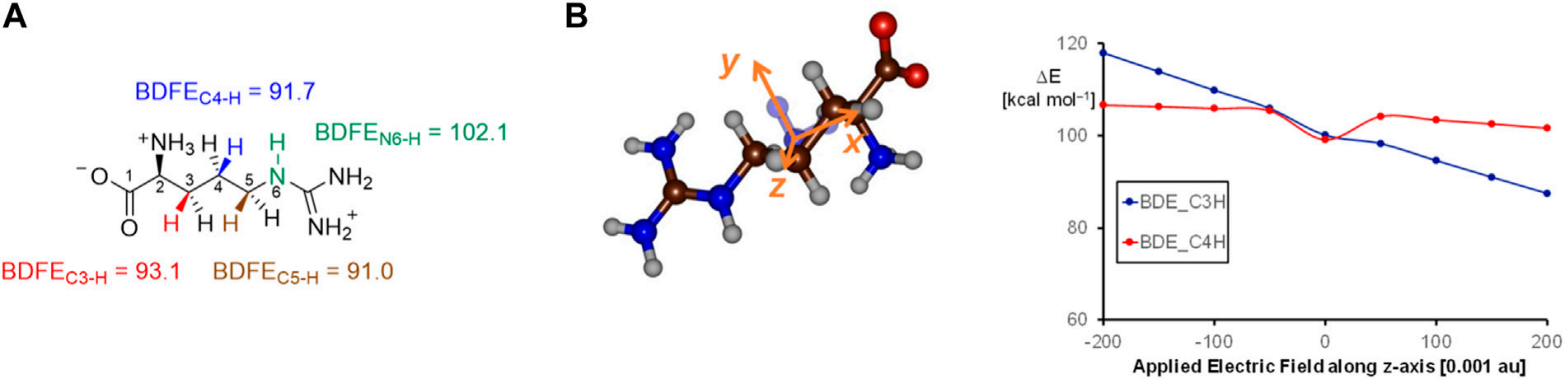
**(A)** Gas-phase bond dissociation free energies of C−H/N−H bonds in an isolated L-Arg molecule calculated at the UB3LYP/6-311+G* level of theory. **(B)** Electric field effects along the molecular *z*-axis and the changes in bond dissociation free energies calculated at the UB3LYP/6-311+G* level of theory.

To understand the fundamental factors of the hydrogen atom abstraction process, we took an isolated L-Arg amino acid and calculated the strengths of various C−H and N−H bonds. Thus, the energy for a hydrogen atom abstraction by an iron(IV)-oxo species is equal to the sum of the C−H bond of the substrate that is broken and the O−H bond of the iron(III)-hydroxo species that is formed (Bordwell and Cheng, 1991; de Visser, 2010; Pegis et al., 2018). These bond dissociation free energies (BDFEs) were calculated as the difference in the energy between the substrate and the sum of a hydrogen atom and the substrate with a hydrogen atom removed. In the gas phase, the C_4_−H and C_5_−H BDFE values (Figure 7A) are of similar energy, namely, ΔG = 91.7 kcal mol^−1^ for the C_4_−H bond and ΔG = 91.0 kcal mol^−1^ for the C_5_−H bond. As such, under ideal circumstances, i.e., without external perturbations, arginine hydroxylation should give a mixture of C_4_-hydroxylation and C_5_-hydroxylation with similar hydrogen atom abstraction barriers. By contrast, the C_3_−H bond has a BDFE of ΔG = 93.1 kcal mol^−1^, and it should be more challenging to cleave the C_3_−H bond than the C_4_−H or C_5_−H bonds. The N_6_−H bond strength was also evaluated and found to be well higher in energy than either of the C−H bonds, and hence, activation of the N−H bond will be challenging.

We then explored whether external perturbations could change the order and bond energies of the various C−H bonds in L-Arg. To this end, we applied an external electric field along the molecular x-, y-, or *z*-axis of the substrate and recalculated the BDFE values for the C_3_−H and C_4_−H bonds, as shown in Figure 7B. Previously, this approach was shown to change the electronic configuration and reactivity patterns of enzymes (Shaik et al., 2004; Shaik et al., 2018; Lin et al., 2021; Gérard et al., 2023). As can be seen, an electric field along the molecular *z*-axis of L-Arg affects the C_3_−H and C_4_−H bond strengths. Thus, this field is along the C−H bonds, but as the hydrogen atoms are on different sides of the substrate, a positive field has a bond-weakening effect for one group and a bond-strengthening effect for the other group. As a result, for large positive electric fields (the field direction is defined as in Gaussian), we observe a weak C_3_−H and strong C_4_−H bond, while with negative fields, the order is reversed. As a consequence, a local electric field, as appears in a protein, can influence the bond strengths of a substrate and guide the reaction toward the weaker of the two bonds to trigger chemo- or regioselectivity.

**FIGURE 8:**
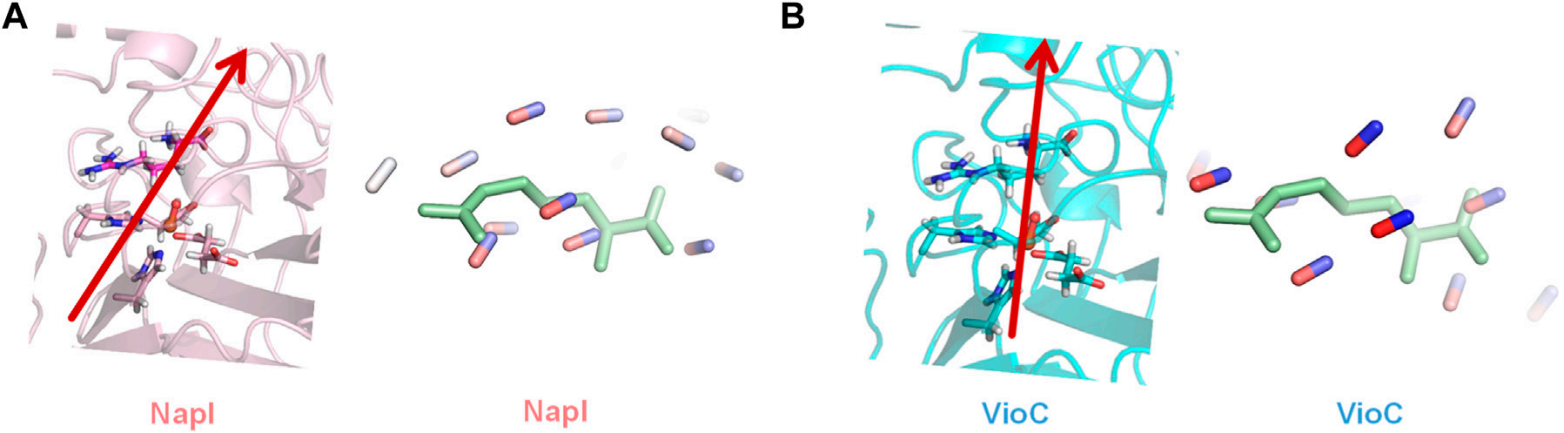
Electric dipole moment (vector in red) and substrate-binding pocket electric field gradients with diatomic sticks with blue representing a positive direction and those with red representing the negative direction. **(A)** Dipole moment and field gradients for NapI. **(B)** Dipole moment and field gradients for VioC.

As the local environment around L-Arg may affect the C−H bond strengths and thereby the reactivity and selectivity, we decided to analyze the active site charge distributions and polarity. We started with investigating the electric dipole moment of the model. These electric dipole moment vectors are shown in red in Figure 8. In VioC, the electric field vector points along the Fe−O bond and the C_3_−H bonds. Therefore, the substrate C−H bond in VioC is aligned with the electric dipole vector and has the C_3_−H bond weakened with respect to other C−H bonds in the substrate. The electric dipole moment in VioC, as a result, guides the reaction to C_3_−H hydrogen atom abstraction and C_3_-hydroxylation. By contrast, in NapI, the electric dipole vector points along the backbone of the substrate and does not seem to influence C−H bond strengths. Indeed, the lowest energy hydrogen atom abstraction barrier is for the C_5_−H hydrogen atom abstraction, which is the weakest C−H bond in the gas phase. Consequently, the environmental effects in NapI do not appear to influence substrate C−H bond strengths, and NapI follows the patterns based on BDFE values of the substrate. An analysis of the electric field gradients of the substrate-bound protein structures comes to the same conclusions, namely, in NapI, the field gradient is along the substrate backbone, while in VioC, it is located along one of the C−H bonds. Overall, these calculations on various L-Arg-activating nonheme iron dioxygenases show that nature has designed proteins very carefully with specific charge distributions and dipole moments that guide the selectivity and specificity of reaction patterns. Furthermore, the insights obtained from the external electric field perturbations can be utilized in biotechnology to engineer proteins and give them novel functions. For instance, engineering of the VioC protein and mutation of Asp_270_ by an Ala residue may change the dipole moment in the protein and trigger electrostatic changes that will enable substrate desaturation, as is seen in the NapI structure.

## 5 Conclusion

Computational modeling on enzymatic reaction mechanisms is reviewed with particular emphasis on L-Arg activation by nonheme iron dioxygenases. Thus, the class of nonheme iron and *α*KG-dependent dioxygenases react efficiently with L-Arg as a substrate, but a large variety of products can be obtained, including 3-hydroxyarginine, 4-hydroxyarginine, 5-hydroxyarginine, and 4,5-dehydroarginine. To understand the product distributions and bifurcation pathways, a series of computational studies was performed using either QM/MM or DFT cluster models. Both approaches match the experiment well and predict low-energy barriers, leading to the experimentally determined products. The calculations show that substrate binding and positioning guide the enzyme toward a specific selectivity. However, the intricate bifurcation pathways are determined by charge distributions within the substrate-binding pocket. Thus, local electric field effects and dipole moments influence the substrate C−H bond strengths and create highly selective enzyme catalysts. It would be interesting to see if proteins can be engineered based on computationally suggested charge distributions and engineered active site structures.
